# Ultrasound-Guided Occipital Nerve Blocks as Part of Multi-Modal Perioperative Analgesia in Pediatric Posterior Craniotomies: A Case Series

**DOI:** 10.3390/children10081374

**Published:** 2023-08-11

**Authors:** Jordan I. Gaelen, Michael R. King, John Hajduk, Angelica Vargas, David J. Krodel, Ravi D. Shah, Hubert A. Benzon

**Affiliations:** Department of Pediatric Anesthesiology, Ann and Robert H. Lurie Children’s Hospital of Chicago, Feinberg School of Medicine, Northwestern University, Chicago, IL 60611, USA

**Keywords:** pediatrics, occipital nerve block, anesthesia, posterior craniotomy, postoperative analgesia, regional anesthesia

## Abstract

Various regional anesthetics have been used for postoperative analgesia for pediatric craniotomy. In this case series, we report retrospectively collected data on postoperative pain and analgesic use in 44 patients who received ultrasound-guided occipital nerve blocks in addition to intravenous analgesic agents for posterior craniotomy procedures. In the immediate post-anesthesia care unit, pain was rated as zero or well controlled in 77% of patients, with only 43% requiring intravenous or demand patient-controlled analgesia opioids. There were no block-related complications. Occipital nerve blocks may constitute a safe and effective component of multimodal analgesia in this population.

## 1. Introduction

Greater occipital nerve blocks are well described and frequently used in the management of pediatric headache disorders, including those related to prior surgery and trauma [1,2]. Descriptions of their use for intraoperative and postoperative pain have been the subject of prior case reports [3,4] and a recent randomized control trial [5]. In order to determine the feasibility of using regional anesthesia for postoperative pain following posterior cranial surgery, we began performing ultrasound-guided greater occipital nerve blocks for these procedures at our institution.

Here, we report postoperative pain and opioid use in 44 patients who received intraoperative greater occipital nerve blocks for posterior cranial surgery.

## 2. Case Description

The study was deemed exempt from review by the Ann and Robert H. Lurie Children’s Hospital of Chicago Institutional Review Board (IRB 2020-3969) as a quality improvement initiative using deidentified, retrospective patient data and granted a full waiver of Health Insurance Portability and Accountability Act (HIPAA) authorization. Informed consent and site marking for greater occipital nerve blocks were performed with a parent or legal guardian per institutional standards. Between August 2016 and October 2019, 44 patients received greater occipital nerve blocks for procedures involving posterior cranial incisions for decompression or resection of tumors, cysts, or cavernous malformations. An anesthesiologist obtained informed consent for the block procedure and performed regional anesthesia site-marking with each patient’s parent or guardian in the preoperative area.

**Figure 1:**
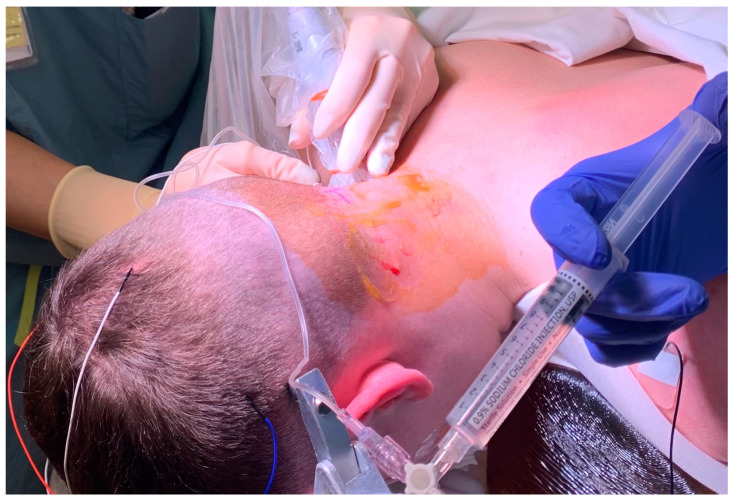
Performance of a right greater occipital nerve block at the C2 level. The probe is lateral and slightly rotated for an in-plane approach.

**Figure 2:**
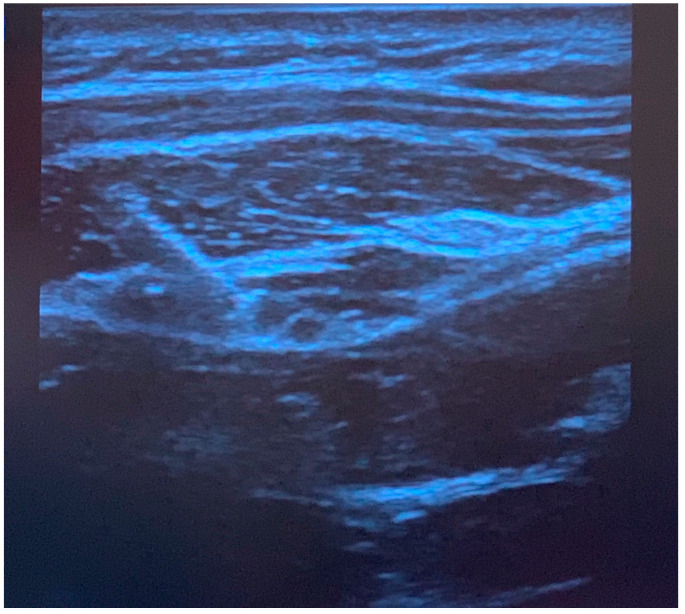
Ultrasound image of greater occipital nerve block injection. The needle is positioned superior to the obliquus capitis inferior muscle, and local anesthetic spread is observed in the fascial plane. The semispinalis muscle is observed above the fascia.

Greater occipital nerve blocks were performed following induction of anesthesia, prone positioning, and sterile preparation. To perform the blocks, a linear ultrasound probe was used to locate the C2 spinous process, and the probe moved laterally and rotated (Figure 1) to find the obliquus capitis inferior muscle plane (Figure 2) as previously described [6]. Blocks were performed with 0.2% ropivacaine, with volume and additives determined by the anesthesiologist. Prior to incision, additional injections of epinephrine with or without local anesthetic were performed at the incision site by the surgical team for hemostasis. Intraoperative analgesic and sedative adjunct usage was determined by the anesthesiologist. Further details of intraoperative characteristics and intraoperative medications can be found in Table 1.

Following surgery, patients were placed supine and extubated prior to transfer to the recovery area. All patients were initiated on patient-controlled analgesia (PCA) with morphine or hydromorphone on a demand-only basis without continuous basal infusion. Pain scores were recorded using the numeric rating scale (NRS), visual analog scale (VAS), revised Faces, Legs, Activity, Cry, Consolability (rFLACC) scale, or FACES scale. We defined pain scores of 0, 1–3, 4–7, and 8–10 as no pain, well-controlled, poorly controlled, and uncontrolled pain, respectively. Patients were transitioned to oral opioids on postoperative day 2, and adjuncts such as non-steroidal anti-inflammatory drugs (NSAIDs) and acetaminophen were added when appropriate by the surgical team. Data on PCA use, pain scores, and adjunct use were collected retrospectively and analyzed.

Our analysis included 44 patients, 18 males and 26 females, who received occipital nerve blocks for posterior fossa procedures. The mean age was 12 years (range: 4–15). The most common indications for surgery were Chiari malformation type I (*n* = 25, 57%) and brain tumor (*n* = 15, 34%). Blocks were performed 215 min (range: 161–262) prior to emergence with 0.18 (interquartile range: 0.13–0.26) mL/kg of 0.2% ropivacaine under ultrasound guidance, with no related untoward events at any point. Additional patient demographic information, intraoperative medications, and block details are listed in Table 1. There were no adverse events related to greater occipital nerve blocks. All blocks were performed with 0.2% ropivacaine without additives, except for one 15 kg patient who received 0.2% ropivacaine with 1 mcg/mL of clonidine (total of 3 mL given for a total of 3 mcg of clonidine).

**Figure 3:**
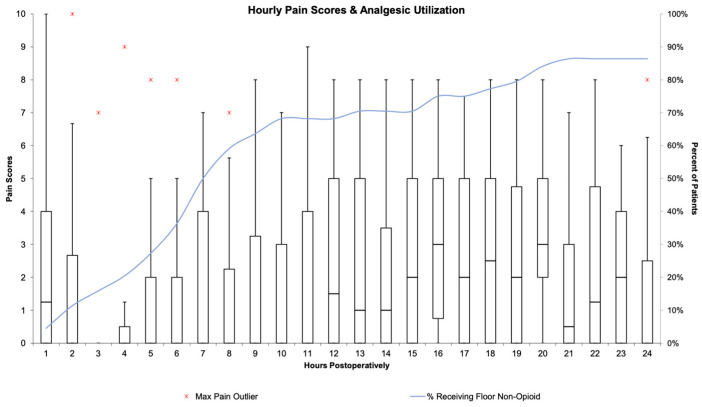
Hourly pain scores and opioid analgesic utilization. Box and whisker plots represent hourly pain scores, scaled on the *Y*-axis labeled on the left. The blue line represents the cumulative percentage of patients receiving non-opioid analgesics, scaled on the *Y*-axis labeled on the right.

In the post anesthesia care unit (PACU), pain was rated as no pain or well-controlled pain in 77% of patients, with 43% receiving intravenous or demand PCA opioids in the earliest period (Table 2). The median patient received zero opioids in the PACU, while of those who received opioids, the median morphine equivalents were 0.0315 mg/kg (interquartile range (IQR): 0.014–0.064). Patients requiring opioids in the PACU were less likely to have received intravenous acetaminophen versus the group at large (44.4% versus 59.1%) and slightly less likely to have received dexmedetomidine (27.8% versus 31.8%). Patients reporting poorly controlled or uncontrolled pain were more likely to have received intravenous acetaminophen versus the group at large (70% versus 59.1%) and less likely to have received dexmedetomidine (20% versus 31.8%). Seventy-five percent of median pain scores remained zero through the 12th post-op hour. Characteristics of postoperative pain and analgesic utilization are presented in Figure 3 and Table 2.

Following surgery, no long-term complications related to blocks were noted at subsequent office visits. One patient reported neck pain without headaches for one week. Another patient reported migraines one week prior to outpatient follow up that resolved without intervention.

## 3. Discussion

Pain control following craniotomy is of significant clinical concern and has been historically undertreated [7]. Studies in adults have shown that up to 60–80% of patients may experience severe pain in the acute post-operative period following craniotomy, and that pain is more frequent and worse than anticipated by the patient [7,8,9]. There are few studies that have examined postoperative pain in the pediatric population, but those that have found that adequate pain relief can be achieved via a multimodal analgesic approach [10,11]. Traditionally, opioids have been used as a mainstay of pain control in this population, but these medications come with potentially significant adverse effects that must be monitored closely postoperatively and may interfere with post-operative neurological exams [9,12]. Nerve blocks or local anesthesia have been shown to be effective components of a multimodal analgesic approach, but most studies limit discussion to scalp blocks [9,12,13].

Scalp blocks offer a potential means for pain control after a craniotomy and have been well studied. A recent randomized, placebo-controlled study on pediatric patients undergoing a craniotomy for brain tumor demonstrated improved postoperative pain and intraoperative hemodynamic stability with scalp blocks using 0.2% ropivacaine [14]. A recent meta-analysis looking at 12 studies demonstrated that scalp blocks could lead to lower pain scores, a longer time to first request for analgesia medications, and fewer pain medications necessary within the first 12 h [15]. However, this meta-analysis only evaluated adult patients and was not specific to the posterior region focused on in this study.

In contrast to scalp blocks, greater occipital nerve blockade may be used for medial posterior fossa surgeries but is of limited value for other craniotomy incisions. However, using a more proximal approach at the C2 level may result in additional blockade of other nearby nerves, such as the lesser occipital and third occipital nerves, which are closer together at this level [6]. The blockade of midline incisions, such as posterior fossa decompression, is also possible via wound infiltration. The use of liposomal bupivacaine has been explored for this purpose and may represent another possible therapy for analgesia for midline posterior incisions [16].

A recent randomized, controlled trial by Nassar et al. from Egypt found greater occipital nerve blocks to be associated with greater duration of postoperative analgesia in a group of 17 patients with American Society of Anesthesiology (ASA) status 1 or 2 undergoing posterior fossa craniotomy compared with 18 controls [5]. In this case series, we present the largest cohort report of greater occipital nerve blocks in pediatric posterior craniotomies to date. Our cohort also expands the practice described by Nassar et al. by including patients with an ASA status of 2 or 3. A large portion of our patients had no pain or well-controlled pain in the immediate and initial period after surgery, with pain scores rising thereafter. Our results are consistent with Nassar et al. and two other studies conducted in adults, in which pain scores were significantly lower than control at the first 4 h period post-craniotomy [5,17,18]. The advantages of greater occipital nerve blockade include ease and safety of the technique, speed of performance (typically 5 to 10 min for bilateral blockade in our experience), and the ability to perform the block in the same position and field as the surgery.

This study’s primary limitations are its relatively small sample size and its lack of a control group, making it difficult to determine the magnitude which the greater occipital blocks reduced opioid use and pain scores. Subjective pain scores were collected from the electronic medical record retrospectively, which introduces the possibility of variation in documentation and assessment quality. As has been described previously, while the NRS, VAS, rFLACC, and FACES scores are well validated, it may still be challenging to accurately assess pain in this population [19]. Variable use of analgesics, such as PCA utilization in the PACU and on the floor, may also have affected reported pain scores. Future prospective studies may be able to control for any variation by implementing a standardized assessment scheme.

In summary, we present a large case series of greater occipital nerve blocks for postoperative pain control in posterior fossa surgery. We observed no adverse events related to the blocks and found them to be easy to learn and perform. A prospective, randomized, placebo-controlled study to quantify reductions in opioid use and pain scores attributable to greater occipital blockade is necessary.

## Figures and Tables

**Table 1:** Patient demographics and intraoperative characteristics.

Patient Demographics	Value (*n* = 44)
Sex, % male	18 (41%)
Age, years	12 (range: 4–15)
Weight, kg (IQR)	35.2 (17.2–52.8)
ASA Physical Status	
2	25 (57%)
3	19 (43%)
Primary diagnosis	
Chiari malformation type I	25 (57%)
Brain tumor	15 (34%)
Other ^a^	4 (9%)
**Intraoperative Characteristics**	
Anesthesia Duration, min	266 (202–304)
Block to Case End Duration, min	215 (161–262)
**Intraoperative Medications**	N (% Receiving); Median dose mg/kg (IQR)
Fentanyl	44 (100%); 3.2 (2.0–4.3)
Dexamethasone	38 (86.4%); 0.16 (0.09–0.28)
Acetaminophen	26 (59.1%); 12.8 (12.5–15.0)
Dexmedetomidine	14 (31.8%); 0.0009 (0.00044–0.0016)
Morphine	3 (7%); 0.072 (0.058–0.088)
**Block Details** ^b^	
Total Bilateral Volume, mL	6 (4–9)
Total Bilateral Volume, mL/kg	0.18 (0.13–0.26)
Block Administration Duration, min	7 (range: 3–12)

^a^: Other primary diagnoses: arteriovenous malformation of brain (*n* = 1), cyst of posterior cranial fossa (*n* = 1), and lesion of posterior fossa (*n* = 2); ^b^: Ropivacaine, 0.2%, ultrasound-guided. Abbreviations: ASA, American Society of Anesthesiologists; IQR, interquartile range.

**Table 2:** Postoperative course and analgesia outcomes.

Postoperative Course	Value (*n* = 44)
PACU Pain Scores	
No Pain (0/10)	30 (68%)
Well Controlled (1–3)	4 (9%)
Poorly Controlled (4–7)	7 (16%)
Uncontrolled (8–10)	3 (7%)
Patients Requiring PACU Opioid	19 (43%)
PACU Morphine Equivalents, mg/kg (IQR)	0 (0–0.025)
PONV	6 (13.4%)
1st 12-h Floor Morphine Equivalents, mg/kg (IQR)	0.06 (0.04–0.13)
2nd 12-h Floor Morphine Equivalents, mg/kg (IQR)	0.07 (0.03–0.16)
PCA Discontinuation	
≤1 Day	30 (73%)
≥1 Day	11 (27%)
Time to Discharge, days (IQR)	3 (2–4)

Abbreviations: PACU, post-anesthesia care unit; IQR, interquartile range; PONV, postoperative nausea/vomiting; PCA, patient-controlled analgesia.

## Data Availability

All data are provided in the paper.
